# Retromer Complex and PI3K Complex II-Related Genes Mediate the Yeast (*Saccharomyces cerevisiae*) Sodium Metabisulfite Resistance Response

**DOI:** 10.3390/cells10123512

**Published:** 2021-12-13

**Authors:** Xuejiao Jin, Huihui Zhao, Min Zhou, Jie Zhang, Tingting An, Wenhao Fu, Danqi Li, Xiuling Cao, Beidong Liu

**Affiliations:** 1 State Key Laboratory of Subtropical Silviculture, School of Forestry and Biotechnology, Zhejiang A&F University, Lin’an, Hangzhou 311300, China; jinxuejiao1991@cau.edu.cn (X.J.); zhaohuihui@stu.zafu.edu.cn (H.Z.); zhoumin@stu.zafu.edu.cn (M.Z.); zhangjie@stu.zafu.edu.cn (J.Z.); antingting@stu.zafu.edu.cn (T.A.); fuwenhao@stu.zafu.edu.cn (W.F.); lidanqi@stu.zafu.edu.cn (D.L.); 2 Department of Chemistry and Molecular Biology, University of Gothenburg, Medicinaregatan 9C, SE-413 90 Goteborg, Sweden; 3 Center for Large-Scale Cell-Based Screening, Faculty of Science, University of Gothenburg, Medicinaregatan 9C, SE-413 90 Goteborg, Sweden

**Keywords:** yeast, Na_2_S_2_O_5_, genome-wide screen, retromer complex, Vps34, PI3K

## Abstract

Sodium metabisulfite (Na_2_S_2_O_5_) is widely used as a preservative in the food and wine industry. However, it causes varying degrees of cellular damage to organisms. In order to improve our knowledge regarding its cyto-toxicity, a genome-wide screen using the yeast single deletion collection was performed. Additionally, a total of 162 Na_2_S_2_O_5_-sensitive strains and 16 Na_2_S_2_O_5_-tolerant strains were identified. Among the 162 Na_2_S_2_O_5_ tolerance-related genes, the retromer complex was the top enriched cellular component. Further analysis demonstrated that retromer complex deletion leads to increased sensitivity to Na_2_S_2_O_5_, and that Na_2_S_2_O_5_ can induce mislocalization of retromer complex proteins. Notably, phosphatidylinositol 3-monophosphate kinase (PI3K) complex II, which is important for retromer recruitment to the endosome, might be a potential regulator mediating retromer localization and the yeast Na_2_S_2_O_5_ tolerance response. Na_2_S_2_O_5_ can decrease the protein expressions of Vps34, which is the component of PI3K complex. Therefore, Na_2_S_2_O_5_-mediated retromer redistribution might be caused by the effects of decreased Vps34 expression levels. Moreover, both pharmaceutical inhibition of Vps34 functions and deletions of PI3K complex II-related genes affect cell tolerance to Na_2_S_2_O_5_. The results of our study provide a global picture of cellular components required for Na_2_S_2_O_5_ tolerance and advance our understanding concerning Na_2_S_2_O_5_-induced cytotoxicity effects.

## 1. Introduction

Sodium metabisulfite (Na_2_S_2_O_5_) is commonly used as a preservative in a variety of food products, wine, and drugs due to its antimicrobial and antioxidant properties [1]. In the process of wine production, it can inhibit the growth of bacteria, control the activities of fermentation microorganisms, and delay the start time of fermentation. It is also conducive to the precipitation of suspended solids in the fermentation substrate. In the process of beer production, it helps to maintain the fresh flavor of beer and cover up the aging flavor [2,3].

When ingested, Na_2_S_2_O_5_ can interact with water and become oxidized to sulfite radicals, initiating molecular oxidation [4]. It has been reported that sulfites cause varying degrees of damage to animals, plants, and microorganisms. Sulfite oxidation leads to increased lipid peroxidation in organs such as the liver and kidneys. Levels of malondialdehyde (MDA), a product of lipid peroxidation, increase significantly in Na_2_S_2_O_5_-treated rat tissues [5]. Oxidative damage and apoptosis induced by Na_2_S_2_O_5_ in other tissues such as lung, brain, and stomach have also been revealed [6,7,8]. Likewise, Na_2_S_2_O_5_ can induce lipid peroxidation and loss of cell membrane integrity in microorganisms [9].

Study also indicated that Na_2_S_2_O_5_ treatment works by increasing xanthine oxidase and xanthine dehydrogenase enzyme activities as well as by exerting endoplasmic reticulum stress [10]. Electrophysiological analysis of the effects induced by Na_2_S_2_O_5_ or sulfur dioxide derivatives on ionic currents in rats suggested that calcium, sodium, and potassium channels are affected [11]. In cardiomyocytes and neurons treated with Na_2_S_2_O_5_, the voltage-gated Na^+^ current was stimulated whereas the K^+^ current was dose-dependently inhibited [12]. In rat heart, Na_2_S_2_O_5_ activates KATP channel expression and reversely inhibit the expression of the L-Ca^2+^ channel [13]. In addition, sulfur dioxide derivatives upregulate intracellular Ca^2+^ levels as they modulate the Na/Ca exchange current [11]. Thus, the effects of Na_2_S_2_O_5_ and its derivatives on cells might be due to their oxidative toxicity and excitotoxicity. In plants, when the absorption rate of sulfur dioxide and its sulfite derivatives is higher than the detoxification rate, photosynthesis and energy metabolism are compromised, resulting in yellowing of leaves and even death of plants in a short time [14].

Investigation into how to limit the addition of harmful and allergenic preservatives in food and wine is an important research field. Moreover, sulfite tolerance in wine yeast is of great interest, as this is an important technological characteristic for winemaking [15]. Yeast uses different strategies such as sulfite reduction, sulfite oxidation, acetaldehyde production, and active sulfite efflux to cope with the toxicity caused by sulfites and other sulfur dioxide derivatives [16]. Ssu1 is the key regulator of sulfite efflux, acting as a sulfite efflux pump to transport sulfite out of the cell [17]. Fzf1, a transcription factor, plays a positive role in Ssu1 transcription [18] and is important for yeast sulfite tolerance. Studies also indicate that autophagy is required for sulfur dioxide tolerance to develop in yeast [19]. Building on the above-mentioned studies, we conducted a genome-wide investigation to provide a global picture of cellular processes that are required for cellular Na_2_S_2_O_5_ tolerance and to also shed light on the mechanism underlying Na_2_S_2_O_5_ toxicity.

Due to its fully characterized genome and diverse mutant collections, yeast provides an effective model system to study toxic mechanisms of chemicals and to identify cellular components required for the corresponding resistance machineries [20,21]. For instance, Hillenmeyer and colleagues teased out the functions of nonessential yeast genes through a high-throughput screen in which they measured the fitness of yeast mutants upon incubation with a variety of chemicals or in various environmental conditions [22]. In order to acquire a systemic understanding of the cytotoxicity and cellular response mechanisms of Na_2_S_2_O_5_, a genome-wide screen of a *Saccharomyces cerevisiae* non-essential deletion collection was performed to identify Na_2_S_2_O_5_-tolerance genes. This screen reproducibly identified 162 Na_2_S_2_O_5_-sensitive mutants and 16 Na_2_S_2_O_5_-tolerant mutants, representing genes that function in Na_2_S_2_O_5_ response. Further characterization of these mutants highlighted the importance of the retromer complex and phosphatidylinositol-3-monophosphate kinase (PI3K) complex II-related proteins in surviving Na_2_S_2_O_5_ challenges. At the same time, our analysis indicated that Vps34 and the retromer complex might be potential targets of Na_2_S_2_O_5_. Our results provide valuable insights into the molecular basis of cellular Na_2_S_2_O_5_ tolerance and might lead to new clues helpful in optimizing yeast strains for wine production as well as sulfite utilization.

## 2. Materials and Methods

### 2.1. Chemicals and Strains

Na_2_S_2_O_5_ was obtained from Sigma-Aldrich (St. Louis, MO, USA, Cat#13459). Anti-Flag and anti-PGK1 antibodies were purchased from Cell Signaling Technology (Boston, MA, USA, Cat#14793) and Abcam (Cambridge, UK, Cat#ab113687), respectively. The methylene blue stain solution was obtained from Solarbio Life Sciences (Beijing, China, Cat#G1180), and PI3K inhibitor AS604850 was obtained from Selleck Chemicals (Houston, TX, USA, Cat#S2681). The widely used laboratory strain BY4741 (*MAT*a *ura3∆0 leu2∆0 his3∆1 met15∆0*) was used for the growth curve measurement with or without Na_2_S_2_O_5_ treatment. The SGA-v2 collection was a gift provided by Charles Boone (Toronto University, Toronto, ON, Canada). The SGA-v2 collection was created from yeast parental strain BY4741. Additionally, each ORF was replaced with a KanMX4 cassette to generate the single deletion mutant. On each 384-arrayed plate in the collection, *his3Δ* (*MAT*a *his3Δ::kanMX4*) at the outer ring was usually used as the SGA control strain. All deletion mutants used in this study were obtained from the SGA-v2 collection except for *vps34∆*. As *vps34∆* was not included in the SGA-v2 collection, we obtained this mutant from the commercial yeast knock out collection (https://horizondiscovery.com/en/gene-modulation/overexpression/non-mammalian/products/yeast-knockout-collection) (accessed on 12 December 2021). The parental strain in this collection is also BY4741, and vps34 ORF was replaced with a KanMX cassette.

Strains containing proteins fused with a green fluorescent protein (GFP) tag at the C-terminus were obtained from the commercial Yeast GFP Clone Collection [23]. For this collection, the genotype of the parent haploid *S. cerevisiae* strain (ATCC201388) is: *MAT*a *his3Δ1 leu2Δ0 met15Δ0 ura3Δ0*. The GFP fusion proteins are integrated into the yeast chromosome through homologous recombination and are expressed using endogenous promoters. More information can be found on the website: https://www.thermofisher.cn/cn/zh/home/references/protocols/proteins-expression-isolation-and-analysis/gfp-protocol/yeast-gfp-clone-collection.html (accessed on 12 December 2021). Strains containing proteins fused with the 5xFlag tag at the *C*-terminus were created from BY4741, which were constructed by genetic manipulation using homologous recombination of overlapping PCR fragments. The plasmid containing the coding sequence of the 5xFlag and LEU2 cassette in our lab was used as a template for overlapping PCR fragment amplification. The primers for homologous recombination are summarized in Table S1. All these constructed strains were verified by DNA sequencing.

### 2.2. Growth Curve Measurement

Pre-cultures were diluted to an OD_600_ of 0.5 in yeast peptone dextrose (YPD) supplemented with 0 mM, 5 mM, 10 mM, 15 mM, and 20 mM of Na_2_S_2_O_5_ (dissolved in ddH_2_O). Cell growth was monitored by a spectrophotometer (Ultrospec 2100 Pro, Biochrom, St. Albans, UK) at different time points, as described previously [24].

### 2.3. Genome-Wide Na_2_S_2_O_5_ Screen and Functional Analysis

The screening was carried out as described previously [24]. Briefly, the SGA-v2 strains were arrayed in 384-format. After cultivated on YPD agar plates (with G418 added), each 384-density array was replicated in quadruplicate to yield a 1536-density array on a single plate containing either no Na_2_S_2_O_5_ or 12 mM Na_2_S_2_O_5_. After incubation for 2 days at 30 °C. Images of the plates were taken and fitness scores of individual mutants was calculated using SGAtools (http://sgatools.ccbr.utoronto.ca/) (accessed on 12 December 2021) [25]. The score was calculated on the basis of normalized colony size for control and Na_2_S_2_O_5_ plates. The experiments were repeated three times. Strains with a cut-off of less than −0.2 in three repeats were selected as mutants that were sensitive to Na_2_S_2_O_5_, and tolerant mutants were selected with a cut-off of more than 0.2 in three repeats. Gene ontology (GO), Kyoto Encyclopedia of Genes and Genomes (KEGG), and physical interactions analysis were performed as described previously [24]. Correlation estimates for all mutants tested in both our screen and previous competition experiment were performed using the OmicStudio tools at https://www.omicstudio.cn/tool (accessed on 12 December 2021).

### 2.4. Spot Test

The spot test was carried out as described previously [23]. Briefly, pre-cultures of different strains were adjusted to an OD_600_ of 0.1 and cultured at 30 °C to reach an OD_600_ of 0.5. Then, cultures were serially diluted and spotted on plates with or without Na_2_S_2_O_5_.

### 2.5. Fluorescence Observation

Log phased cultures (at an OD_600_ of 0.5) of GFP fusion strains were treated with 0, 10, and 15 mM Na_2_S_2_O_5_, respectively, for 3 h. Then, the localizations of GFP proteins were observed using a Zeiss Axio Observer 7.

### 2.6. Western Blot

Pre-cultures were diluted to an OD_600_ of 0.1 and cells were allowed to grow to reach an OD_600_ of 0.5 in YPD before being treated with 0 mM or 15 mM Na_2_S_2_O_5_ for 3 h. Then, the cells were harvested and treated with 0.2 M NaOH for 10 min at room temperature, followed by boiling in HU buffer (200 mM phosphate buffer, pH 6.8, 8 M urea, 5% *w*/*v* SDS, 1 mM EDTA, 100 mM DTT, bromophenol blue) for 10 min for Western blot analysis. The Vps35-GFP, Vps5-GFP, Vps17-GFP, Vps26-GFP, and Vps34-GFP protein levels were detected with rabbit anti-Flag antibody and HRP-conjugated anti-rabbit IgG as the secondary antibody. Pgk1 were detected with mouse anti-PGK1 antibody and HRP-conjugated anti-mouse IgG as the secondary antibody. Pgk1 served as the loading control and bands of Vps34 protein were quantified using ImageJ software.

### 2.7. Methylene Blue Staining

Methylene blue staining was carried out according to the manufacturer’s protocol (Solarbio Life Sciences, Beijing, China). Briefly, the harvested yeast cultures were suspended in 0.1% methylene blue solution and inoculated for 5 min. Then, the cells were observed by a Zeiss Axio Observer 7. The dead cells were stained blue, whereas living cells were colorless.

### 2.8. Use of PI3K Inhibitor

Pre-cultures were diluted to an OD_600_ of 0.5 in YPD supplemented with 10 mM Na_2_S_2_O_5_ and different concentrations of the PI3K inhibitor AS604850. The inhibitor was dissolved in DMSO and the same amount of DMSO was added in all groups. Then, the cell growth was monitored by a spectrophotometer (Biochrom, Ultrospec 2100 Pro).

## 3. Results and Discussion

### 3.1. Determination of Na_2_S_2_O_5_ Concentration for Yeast Genome-Wide Screening

**Figure 1:**
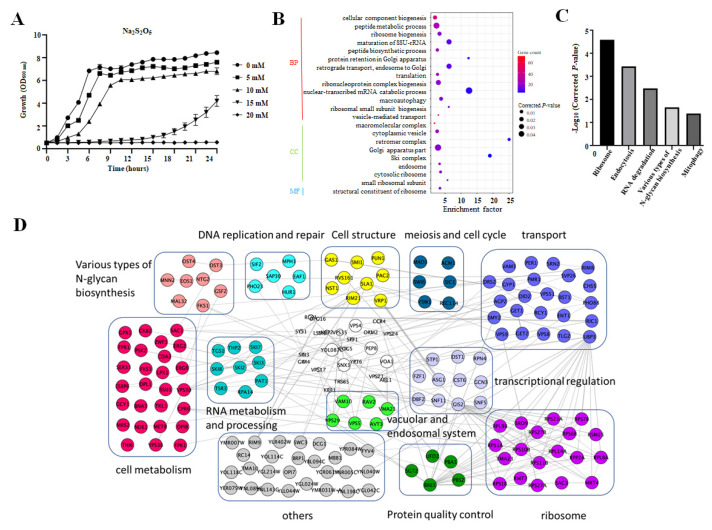
Identification of Na_2_S_2_O_5_-sensitive mutants by genome-wide screen. (**A**) Growth curve of BY4741 in YPD medium with different concentrations of Na_2_S_2_O_5_. (**B**) GO term analysis for the 162 genes for which deletion results in Na_2_S_2_O_5_ sensitivity. BP, biological process; CC, cellular component; MF, molecular function. (**C**) KEGG analysis for the 162 genes for which deletion results in Na_2_S_2_O_5_ sensitivity. (**D**) Physical interactions of the 162 Na_2_S_2_O_5_-sensitive genes. These genes were classified into 12 groups. The node colors indicate different functions. Genes in the white group were not in our list.

In order to determine a proper concentration of Na_2_S_2_O_5_ for the genome-wide screening (enough to give a moderate inhibition effect on growth, usually 50% and enable the detection of both sensitive and tolerant phenotypes), we monitored the growth of BY4741 in YPD medium containing different concentrations of Na_2_S_2_O_5_. From the growth curves, we could see that the wild-type strain in YPD medium without Na_2_S_2_O_5_ had a short adaptation time and could quickly enter the logarithmic phase. However, when Na_2_S_2_O_5_ was added (5 mM and 10 mM), the cells required a longer adaptation time. This inhibition increased with increasing concentration. Moreover, the maximum cell density/yield after reaching the stationary phase also decreased (Figure 1A). When the concentration was increased further (to 15 mM Na_2_S_2_O_5_), the inhibition of yeast growth was significantly enhanced, when increased to 20 mM Na_2_S_2_O_5_, cell growth was completely inhibited (Figure 1A). Cell growth on randomly selected yeast plates in solid YPD medium containing different concentrations of Na_2_S_2_O_5_ were also detected. Replications were carried out in quadruplicate for the SGA control strains (*MATa his3∆::kanMX4*) at the outer ring of the plates and for each single-deletion mutant. Both 10 mM and 12 mM Na_2_S_2_O_5_ resulted in an inhibition on control strain growth of about 50% compared with that of 0 mM Na_2_S_2_O_5_ (Figure S1), but some deletion mutants exhibited notably sensitive phenotypes on the 12 mM Na_2_S_2_O_5_ plate (Figure S1, red circles). At the other end of the spectrum, 14 mM Na_2_S_2_O_5_ inhibited the growth of the control strain too strongly. Thus, considering the growth both in liquid medium and on solid plates, we chose 12 mM as the working concentration for the screening, since it had a clear inhibition effect on the control strain, and at the same time enabled the identification of deletion mutants with a sensitive phenotype.

### 3.2. Genomic Screens of S. cerevisiae Deletion Mutants Revealed Important Cellular Processes Involved in Na_2_S_2_O_5_ Stress

To gain a better understanding of the genes or pathways that are involved in yeast cells’ tolerance to Na_2_S_2_O_5_, a genome-wide screen was performed using the yeast single deletion collection, SGA-v2 [26]. Deletion mutants with more than 4000 nonessential genes were screened on YPD plates with or without 12 mM Na_2_S_2_O_5_, followed by calculation of the growth changes for each mutant using SGAtools. After three independent replicates, a total of 162 mutants with increased sensitivity were identified, representing 162 genes that are involved in tolerance to Na_2_S_2_O_5_ (Table S2). Moreover, a total of 16 mutants with increased tolerance to Na_2_S_2_O_5_ were also identified (Table S3). The information of all deletion mutants tested in this screen along with their fitness scores can be found in Table S4. In order to clarify the functions of genes involved in Na_2_S_2_O_5_ tolerance, we then performed a GO enrichment analysis of genes deleted in Na_2_S_2_O_5_ sensitive mutants using GO Term Finder [24]. We found that gene deletions in strains with increased sensitivity are enriched in various groups. For the biological process category, “maturation of SSU-rRNA”, “protein retention in Golgi apparatus”, “retrograde transport”, “endosome to Golgi”, “nuclear-transcribed mRNA catabolic process”, and “ribosomal small subunit biogenesis” were the main enriched groups. For the cellular component category, “retromer complex”, “Ski complex”, and “small ribosomal subunit” were found to be enriched. In terms of molecular function, only “structural constituent of ribosome” was enriched (Figure 1B). We also conducted a KEGG analysis of the sensitive strains [24], and five enriched pathways were identified including “ribosome”, “endocytosis”, “RNA degradation”, “various types of *N*-glycan biosynthesis”, and “mitophagy” (Figure 1C). In summary, from the GO and KEGG analysis, we can see that transport-, ribosome-, RNA metabolism-, and autophagy/mitophagy-related genes were the main genes that responded to Na_2_S_2_O_5_. In addition, functional classification and physical interaction analysis of these 162 genes were performed as previously described [24]. This analysis demonstrated that the encoded products of over 50% of these genes have physical interactions with each other (Figure 1D). This indicates that Na_2_S_2_O_5_ could induce extensive intracellular responses and these processes or components might work synergistically to confer resistance to Na_2_S_2_O_5_.

In our screen, *ssu1∆* and *fzf1∆* mutants displayed significant growth defects on the Na_2_S_2_O_5_ plates in comparison with the control strains (Table S2). These mutants were reported previously to have an increase in sensitive phenotype [17,18], and they served as positive controls to confirm the reliability of our screening results. In a recent study, a whole-genome quantitative fitness analysis using competition experiments in a yeast knockout collection identified 552 genes that, when deleted, results in increased sensitivity to sulfites [19]. Among these, 98 mutants showed a logFC below −5. We then compared the sensitive mutants between our results and the ones identified by Valero et al. First, we compared the 162 mutants identified from our work with the 98 mutants showing a logFC below −5 in the Valero et al. study. We found 19 mutants that were identified in both studies (Figure S2A). When we compared the 162 mutants with the 552 mutants showing a logFC below −3, 43 mutants were identified in both screens (Figure S2B). The overlapping genes were found to function in diverse processes including vesicular transport, autophagy, and the endosome system. In order to better compare our results with previous competition experiments, correlation estimate for all mutants tested in both studies (4070 mutants) was performed. As shown in Figure S2C, strains in “No.3” group represent the mutants that did not exhibit obvious growth changes in both studies. On the contrary, strains in “No. 5” and “No. 6” groups represent the mutants that deserve more attention as their growth differences are more pronounced in both studies. Strains in “No. 1” and “No. 2” groups represent the mutants that have conflicting results in the two studies. The main reason for this difference could be the screening approach used in our work, which measured growth phenotypes of each mutant individually rather than measuring the competition growth outcomes from a mixed pool of all mutants. Moreover, the treatment conditions and strain background are not exactly the same. Which group the mutant belongs to is shown in Table S5. Taken together, our results highlighted the importance of vesicular transport, ribosomes, RNA metabolism, mitophagy, and endosome-related processes in response to Na_2_S_2_O_5_.

We also performed the GO enrichment and KEGG analysis for the genes deleted in mutants with increased tolerance to Na_2_S_2_O_5_. As shown in Figure S3A, these genes are mainly enriched in “cellular response to DNA damage stimulus”, “DNA repair”, and “mitochondrion”. Additionally, KEGG analysis indicates that pyruvate metabolism-, biosynthesis of antibiotics-, and carbon metabolism-related genes are the negative regulators of Na_2_S_2_O_5_ tolerance (Figure S3B). These genes may be the targets responsible for the Na_2_S_2_O_5_ metabolism and cytotoxicity.

### 3.3. The Retromer Complex Is Required for Yeast Na_2_S_2_O_5_ Tolerance

From the cellular component enrichment analysis, we identified the retromer complex as the top enriched cellular component, with an enriched factor of 25 (Figure 1B). The retromer complex is a type of protein-sorting machinery that is important in the recycling of cargo molecules from the endosome to the trans-Golgi network or the plasma membrane [27,28]. In yeast, the retromer complex consists of two subcomplexes: the cargo selective subcomplex (CSC) and a sorting nexin (SNX) dimer. The CSC is a trimer made up of Vps26, Vps35, and Vps29, and is essential for cargo selection and sorting on the endosomal membrane. The SNX dimer is composed of Vps5 and Vps17 (both contain BAR domains), which are important for membrane remodeling [27,28]. Another sorting nexin adaptor protein, Snx3, also interacts with the CSC to regulate the trafficking of the iron transporter Fet3-Ftr1 heterodimer, Kex2, Ste13, and Pep12 [29,30,31]. In our results, deletion of four of these genes (VPS26, VPS5, SNX3, and VPS17) conferred enhanced sensitivity to Na_2_S_2_O_5_ on the cells (Table S2). Thus, we next focused on investigating the function of this vesicular transport-related complex in Na_2_S_2_O_5_ tolerance. 

**Figure 2:**
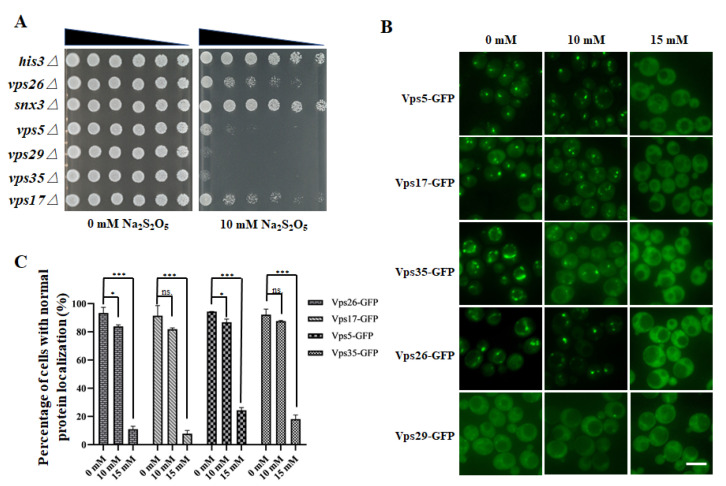
The retromer complex is important for yeast tolerance to sodium metabisulfite. (**A**) Spot-test assays of the SGA control strain (*MATa his3∆::kanMX4*) and retromer deletion mutants. (**B**) Localization of the retromer complex proteins. Scale bar represents 5 μm. (**C**) Quantifications of cells with normal protein localization. Three independent biological experiments were carried out, and for each replicate, a minimum of 200 cells were counted. The vertical axis represents the percentage of cells with normal protein localization, and the horizontal axis represents the different concentrations of Na_2_S_2_O_5_. Error bars indicate standard error. ns., no significant difference; ***, *p* < 0.001; *, *p* < 0.05; Student’s *t*-test.

To determine whether the retromer complex was indeed involved in cellular resistance to Na_2_S_2_O_5_ toxicity, spot-test assays of the four mutants identified in the screen, together with strains containing deletions in other genes in the retromer complex, such as VPS35 and VPS29, were performed, as described previously [24]. The results of these spot-test assays revealed that the growth of these six mutants with retromer complex-related gene deletions was similar to that of the control strain (*his3∆*) on the medium without Na_2_S_2_O_5_, whereas the *vps26∆*, *vps5∆*, *vps29∆*, *vps35∆*, and *vps17∆* mutants exhibited significantly compromised growth under 10 mM Na_2_S_2_O_5_ treatment when compared with the control strain (*his3∆*) (Figure 2A). Therefore, the CSC trimer (Vps26-Vps35-Vps29) and the SNX dimer (Vps5-Vps17) in the retromer complex were deemed important for yeast tolerance to Na_2_S_2_O_5_.

Considering the important roles of the retromer complex in the homeostasis of diverse transmembrane proteins at the plasma membrane and within the endosomal/lysosomal system [27], we suspected that enhanced sensitivity of retromer-related deletion mutants to Na_2_S_2_O_5_ might be attributed to defects in the recycling of key cargo proteins that are required for Na_2_S_2_O_5_ tolerance. Alternatively, the sensitivity could be attributed to a more general disruption of endosome-related or lysosome-related membrane homeostasis induced by retromer protein deletion, such as mitophagy disruption. In the KEGG analysis, mitophagy was a critical pathway that was required for Na_2_S_2_O_5_ tolerance (Figure 1C). Therefore, as mitophagy depends on the functions of the lysosome, retromer complex-related deletion mutants might exhibit mitophagy misfunction, which is not conducive to survival in Na_2_S_2_O_5_. The detailed functional mechanisms utilized by the retromer complex in Na_2_S_2_O_5_ tolerance need to be further investigated. Moreover, unlike Vps5 and Vps17, deletion of SNX3 did not result in any obvious growth defect (Figure 2A). As sorting nexins lead to retromer complex diversity through mediating distinct cargo recognition and retrieval pathways from yeast endosomes, it is thought that yeast Na_2_S_2_O_5_ tolerance mainly depends on the function of the SNX-SAR-retromer but not the SNX3-retromer.

### 3.4. Na_2_S_2_O_5_ Treatment Can Cause Changes in the Localization of the Retromer Complex

Considering the essential roles of the retromer complex in Na_2_S_2_O_5_ tolerance, we speculated that Na_2_S_2_O_5_ might cause cytotoxicity through influence of the functions of this complex. According to the data in the Yeast GFP Fusion Localization Database (https://yeastgfp.yeastgenome.org/) (accessed on 12 December 2021), proteins in the retromer complex are localized on the endosome or the vacuolar membrane [23]. In order to investigate whether Na_2_S_2_O_5_ treatment had any effect on the retromer complex, fluorescence microscopy was used to observe the localization changes of the retromer complex-related proteins (Vps26, Vps29, Vps35, Vps5, and Vps17) after Na_2_S_2_O_5_ treatment, which is a prerequisite for normal retromer functions. Strains with chromosomal C-terminal GFP-tagged proteins were obtained from the commercial Yeast GFP Clone Collection (www.invitrogen.com/clones) (accessed on 12 December 2021) [23]. Vps26, Vps5, and Vps17 were mainly localized on the endosome, and in our study, under normal conditions, Vps26-GFP, Vps5-GFP, and Vps17-GFP were accumulated in bright punctate structures as expected [32]. When exposed to 10 mM Na_2_S_2_O_5_, their localization did not seem to change. However, under 15 mM Na_2_S_2_O_5_ treatment, their localization appeared to be altered (Figure 2B). Likewise, Vps35 was reported to localize on the endosome and the vacuolar membrane [23], and, consistent with this, Vps35-GFP localized to bright puncta and vacuolar rim-like structures in the medium without Na_2_S_2_O_5_. However, when treated with 15 mM Na_2_S_2_O_5_ medium, Vps35-GFP was mislocalized to the cytoplasm (Figure 2B). The localization of Vps29-GFP was ambiguous under normal conditions [23], and thus it was difficult to discern the changes in its localization (Figure 2B). We quantified the cells with normal protein localization before and after Na_2_S_2_O_5_ treatment, and found that more than 90% of the cells displayed normal retromer complex localization in YPD medium (Figure 2C). When exposed to 10 mM Na_2_S_2_O_5_, the proportion of cells with correct protein localization did not change or only slightly changed; while subjected to 15 mM Na_2_S_2_O_5_, about 80% of the cells displayed mislocalized protein (Figure 2C). This pattern was reminiscent of the growth curve in Na_2_S_2_O_5_, in which, when growing in 10 mM Na_2_S_2_O_5_, only slight changes were observed, whereas when growing in 15 mM Na_2_S_2_O_5_, dramatic changes were displayed (Figure 1A). Alterations in retromer complex localization seemed to be correlated with dramatic growth defects in the yeast when exposed to 15 mM Na_2_S_2_O_5_, which implies that defects in cell growth might be due to the mislocalization and potential misfunction of the retromer complex.

In order to verify whether the localization defect of retromer complex-related proteins was induced by reduced protein expression levels, strains containing GFP tagged proteins were detected by anti-GFP antibody. However, the bands of these GFP-tagged proteins were very weak or cannot be detected. In order to clearly show the differences of expression changes, strains containing proteins fused with 5xFlag tag at the *C*-terminus were constructed, followed by protein expression level detection by anti-Flag antibody. As shown in Figure S4A, no significant expression changes in Vps35-5xFlag, Vps26-5xFlag, Vps5-5xFlag, and Vps17-5xFlag were observed before or after Na_2_S_2_O_5_ treatment. Therefore, changes in localization of these four proteins were not caused by decreased protein expression. Moreover, to rule out the possibility that cell death induces mislocalization of retromer complex proteins, the mortality of yeast cells treated with 10 mM and 15 mM Na_2_S_2_O_5_ was observed by methylene blue stain, and the dead cells quantified. The cell mortality of yeast cells treated with 10 mM Na_2_S_2_O_5_ for 3 h was about 22%, and that of 15 mM Na_2_S_2_O_5_ was about 23%, both of which were similar to that of 0 mM Na_2_S_2_O_5_ (18%) (Figure S4B). Since the percentage of protein localization abnormalities under the 15 mM Na_2_S_2_O_5_ treatment was much higher than that of the cell mortality, this suggested that Na_2_S_2_O_5_-induced abnormalities of localization of retromer complex-related proteins were not caused by cell death.

### 3.5. Phosphatidy-linositol-3-monophosphate Is a Potential Signaling Molecule Mediating the Yeast Na_2_S_2_O_5_ Tolerance Response

Evidence has revealed that localization of retromer complex proteins is correlated with phosphatidylinositol-3-monophosphate (PI3P) contents on the endosome membrane [32,33]. PI3P, the phosphorylated derivative of phosphatidylinositol (PI), is enriched on the early endosome, internal vesicles of multivesicular endosomes, vacuole, and autophagosomes [34,35,36]. Vps5 and Vps17 are sorting nexins that contain a Phox homology (PX) domain which is a PI3P-binding module [37]. Thus, the SNX dimer (Vps5 and Vps17) and many other proteins require PI3P-binding for membrane recruitment [33,38]. Moreover, SNX dimer could further enhance the endosome membrane binding efficiency of CSC complex (Vps26-Vps35-Vps29) when CSC is in a low concentration [33]. Therefore, we suspect that the changes in localization of retromer proteins induced by Na_2_S_2_O_5_ might be related to the alteration of endosomal PI3P levels. 

**Figure 3:**
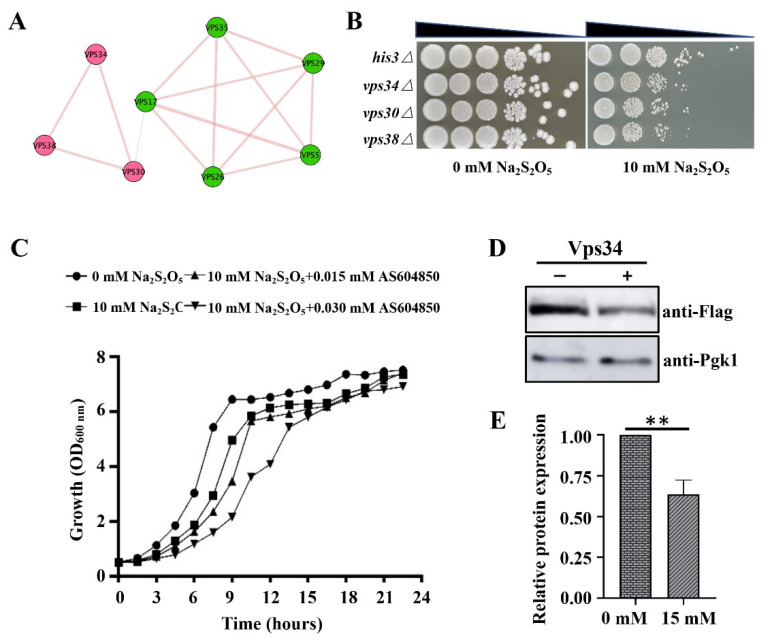
PI3K complex II-related proteins mediate the yeast sodium metabisulfite resistance response. (**A**) Physical interaction between the retromer complex and PI3K complex II. (**B**) Spot test assays of the SGA control strain (*MATa his3∆::kanMX4*) and PI3K complex II-related gene deletion mutants. (**C**) Growth curve of yeast cells cultivated in different media. (**D**) Western bolt analysis of Vps34 expression before and after 15 mM Na_2_S_2_O_5_ treatment (3 h). Pgk1 served as the loading control. (**E**) Accumulation levels of Vps34 were quantified using ImageJ software. Error bars indicate standard error. **, *p* < 0.01, Student’s *t*-test.

Interestingly, in our list of gene candidates, we found another two genes in which deletion resulted in enhanced Na_2_S_2_O_5_ sensitivity: VPS30 and VPS38 (Table S2). Vps30 and Vps38 protein function as subunits of Vps34 phosphatidylinositol 3-kinase (PI3K) complexes. Vps34 is the sole PI3K in yeast that is responsible for the synthesis of PI3P from PI [39]. Vps30 is a subunit of complex I and II, and is important for both autophagy and vacuolar protein sorting. Vps38 is a part of complex II and is only required for vacuolar protein sorting [40]. In addition, a previous study has indicated that PI3K complex II was important for the synthesis of a specific endosomal pool of PI3P which is required for recruitment of the retromer complex [32]. Furthermore, physical interaction analysis demonstrated that the retromer complex interacts with PI3K complex II through Vps17 (Figure 3A). Therefore, we suspect that mislocalization of retromer complex proteins might be related to PI3K complex II misfunction. 

First, we intended to validate the results of our genome-wide screen, which suggested that PI3K complex II-related gene deletions conferred increased sensitivity to Na_2_S_2_O_5_, so spot-test assays of *vps30∆*, *vps38∆*, and *vps34∆* were performed. As shown in Figure 3B, deletion of any of these genes indeed results in compromised yeast growth on plates with Na_2_S_2_O_5_. Therefore, our results suggested that Vps34, Vps30, and Vps38 are important for yeast tolerance to Na_2_S_2_O_5_, and they hint to some extent that cells need sufficient endosomal PI3P synthesis during a Na_2_S_2_O_5_ challenge. 

To obtain further evidence on the central roles of endosomal PI3P on Na_2_S_2_O_5_ tolerance, AS604850, a chemical inhibitor of Vps34, was used to inhibit PI3P synthesis through disruption of Vps34 functions [41,42]. We found that after inhibition of PI3P synthesis by adding AS604850, yeast showed a significantly increased sensitivity to Na_2_S_2_O_5_ in comparation with the group with no added inhibitor (Figure 3C). Taken together, these results indicate that the PI3K complex II-related genes VPS30, VPS38, and VPS34 were needed for yeast to survive in Na_2_S_2_O_5_, and that PI3P is a potential signaling molecule mediating the yeast Na_2_S_2_O_5_ tolerance response. 

To further clarify whether retromer complex mislocalization was induced by disruption of PI3K complex II and endosomal PI3P synthesis, we measured the protein expression levels of Vps34, the core member of PI3K complex II, before and after Na_2_S_2_O_5_ exposure. Strains containing Vps34-5xFlag were constructed by homologous recombination. We found that after 15 mM Na_2_S_2_O_5_ treatment for 3 h, the protein expression of Vps34-5xFlag was reduced by about 65% (Figure 3D,E). Therefore, Na_2_S_2_O_5_-mediated retromer complex redistribution might occur as a result of changes in the amount of PI3P on the endosome that were induced by decreased Vps34 expression levels.

## 4. Conclusions

Na_2_S_2_O_5_ is widely used as a preservative in our daily life, but the specific toxic mechanism and cell responses to it are still unclear. In this study, a genome-wide screen of an *S. cerevisiae* gene deletion library was performed to systemically investigate the mechanisms of Na_2_S_2_O_5_ toxicity and the cellular responses to it. Through the genome-wide screen, a total of 162 mutants deficient for growth when exposed to Na_2_S_2_O_5_ were identified, providing a global picture of intracellular pathways and components required for Na_2_S_2_O_5_ tolerance. Spot-test assays demonstrated the importance of retromer complex and PI3K complex II components in Na_2_S_2_O_5_ tolerance. Further work revealed that retromer and Vps34 might be the targets of Na_2_S_2_O_5_ since we found that the retromer localization was altered and Vps34 protein level was reduced upon Na_2_S_2_O_5_ exposure. The cytotoxic effects of Na_2_S_2_O_5_ may be associated with reduced Vps34 protein accumulation, resulting in decreased PI3P on the endosome membrane, and subsequent changes in retromer complex localizations and functions, which could induce further cell dysfunction.

Taken together, our findings highlight the importance of retromer complex-related and PI3K complex II-related proteins in Na_2_S_2_O_5_ tolerance, and indicate that decreased Vps34 protein accumulation and retromer complex mislocalization are the main cellular effects that caused by Na_2_S_2_O_5_ toxicity. Further investigation regarding the detailed mechanism of how retromer complex functions in Na_2_S_2_O_5_ tolerance will have potential implications in industrial strain design and might influence the utilization of Na_2_S_2_O_5_ in industry.

## Data Availability

The data presented in this study are available on request from the corresponding author.

## Supplementary Materials

The following are available online at https://www.mdpi.com/article/10.3390/cells10123512/s1, Figure S1: Photographs of randomly selected plates in the absence or presence of Na_2_S_2_O_5_, Figure S2: Overlapping mutants identified both in our screen and in previous competition experiments, Figure S3: Functional analysis for the 16 genes deleted in Na_2_S_2_O_5_ tolerant strains, Figure S4: Changes in protein expression and cell mortality rate are not determinants of retromer complex mislocalization, Table S1: The primers for the homologous recombination, Table S2: The 162 genes corresponding to Na_2_S_2_O_5_-sensitive mutants identified from the genome-wide screen, Table S3: The 16 genes corresponding to Na_2_S_2_O_5_-tolerant mutants identified from the genome-wide screen, Table S4: The information of all deletion mutants tested in the genome-wide screen, Table S5: The information of the deletion mutants in the correlation analysis.
