# Supplementary material for: Retromer Complex and PI3K Complex II-Related Genes Mediate the Yeast (Saccharomyces cerevisiae) Sodium Metabisulfite Resistance Response
Source: Cells. 2021 Dec 13;10(12):3512. doi: 10.3390/cells10123512 (PMC8699849; doi:10.3390/cells10123512)
Supplement: Supplementary file 1 [file cells-10-03512-s001.zip › cells-1494403-supplementary.pdf]

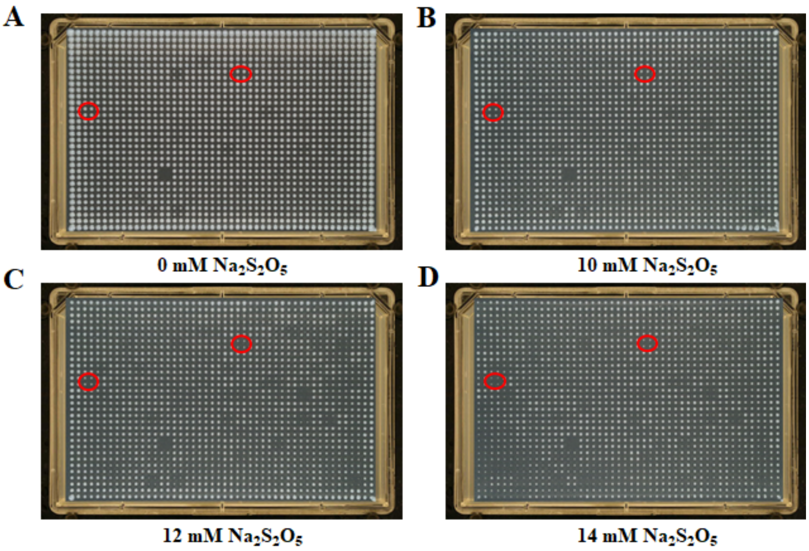

Figure S1. Photographs of randomly selected plates in the absence or presence of $Na_2S_2O_5$. A randomly selected plate in SGA-v2 collection was pinned onto solid YPD medium without (A) or with 10 mM (B), 12 mM (C), or 14 mM (D) $Na_2S_2O_5$. The photograph depicts a set of 384 mutants; each mutant was replicated in quadruplicate to create a 1536-density array. The small colonies marked with red circles are representatives of $Na_2S_2O_5$-sensitive mutants. YPD, Yeast Peptone Dextrose.

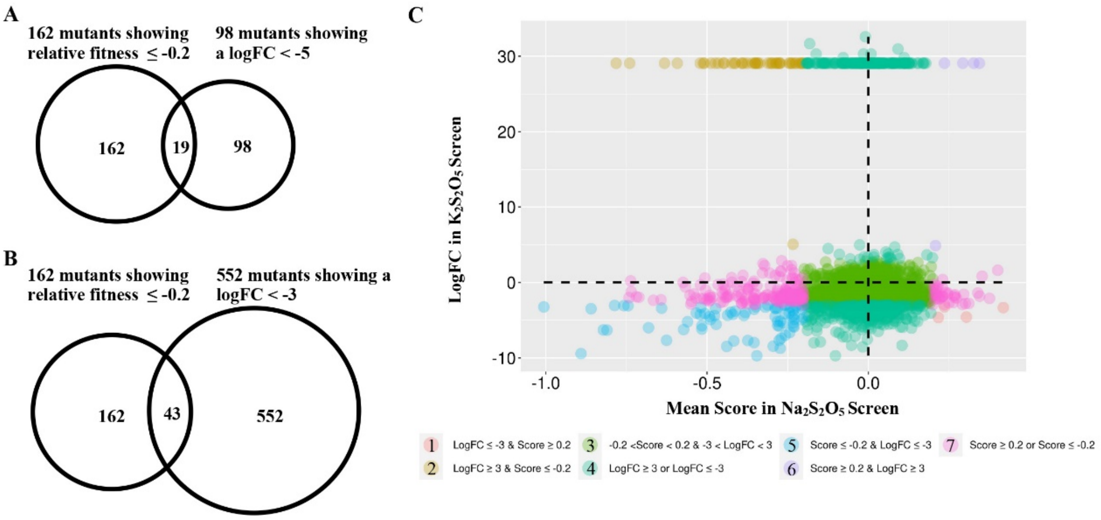

Figure S2. Comparative analyses of our screen and previous competition experiments. (A) The 162 mutants in our list included 19 mutants belonging to a set of 98 mutants showing a logFC below -5 in previous competition experiments. (B) The 162 mutants in our list included 43 mutants belonging to a set of 552 mutants showing a logFC below -3 in previous competition experiments. (C) Correlation analysis of two studies. The vertical axis represents LogFC of each mutant in previous competition experiments. The horizontal axis represents mean score of each mutant from three replicates in our screen. Each circle represents one mutant. Different colors represent different groups with certain classification features.

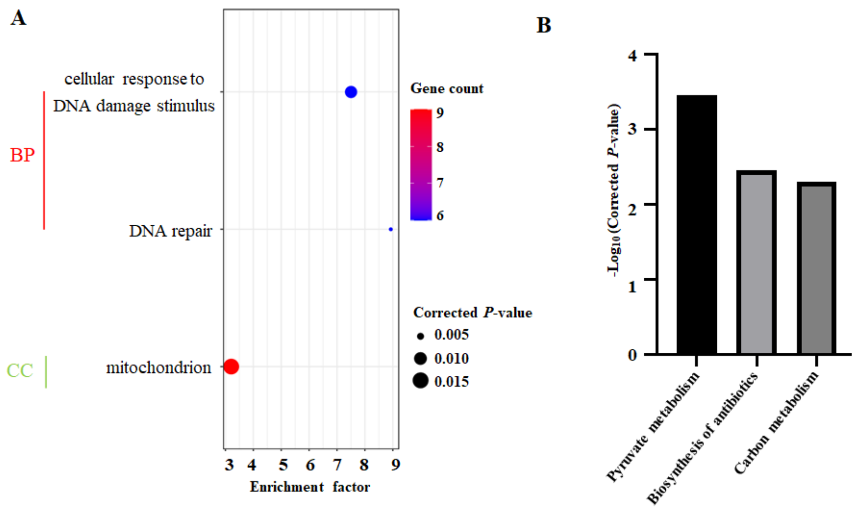

Figure S3. Functional analysis for the 16 genes deleted in $Na_2S_2O_5$ tolerant strains. (A) GO term analysis for the 16 genes for which deletion results in $Na_2S_2O_5$ tolerance. BP, biological process; CC, cellular component. (B) KEGG analysis for the 16 genes for which deletion results in $Na_2S_2O_5$ tolerance.

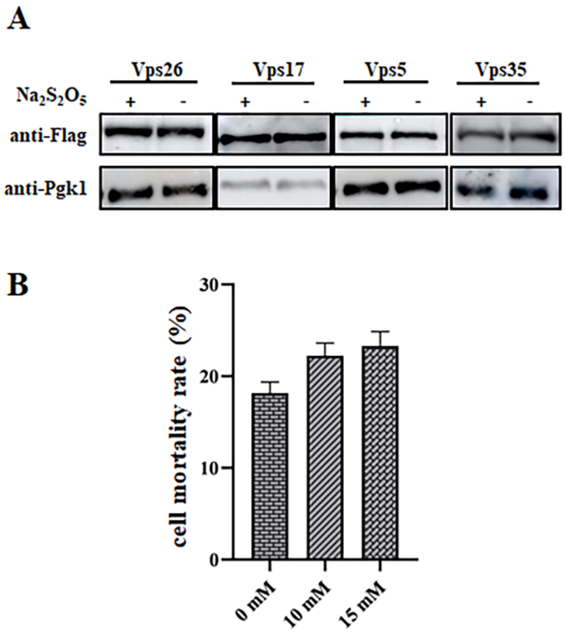

Figure S4. Changes in protein expression and cell mortality rate are not determinants of retromer complex mis-localization. (A) Western bolt analysis of Vps26-5xFlag, Vps17-5xFlag, Vps5-5xFlag, and Vps35-5xFlag before and after 15 mM $Na_2S_2O_5$ treatment (3 h). Pgk1 served as the loading control. (B) Quantifications of dead cells. Three independent biological experiments were carried out, and for each replicate, a minimum of 200 cells were counted. Error bars indicate standard error.

Table S1. The primers for the homologous recombination

Primers	Sequence (5'-3') [a]
VPS5-5xFlag-Leu-1	*CTCAAAAAGAATGCATCGAGCTTTGGGAGACATTCTACCAAACCAATCTT*gactataaggatgatgacgacaaggaattc
VPS5-5xFlag-Leu-2	*TTCATAAATCCTGAGGAACGTGACACATAAAGTTATTGTATACAGATCAT*ttaagcaaggattttcttaacttcttcggc
VPS26-5xFlag-Leu-1	*GATGGCAGAAGATATTTTAAACAATCAGAAATAACATTGTACAGGACCCGG*gactataaggatgatgacgacaaggaattc
VPS26-5xFlag-Leu-2	*GTTGAAAGAACAGAGAACCACATCTTCACCTTATTTAAGGTCGAGCTTTT*ttaagcaaggattttcttaacttcttcggc
VPS29-5xFlag-Leu-1	*TTATACGTTAATGGAGAAGTGAAGGTCGATAAAGTGGTTTATGAAAAGGAA*gactataaggatgatgacgacaaggaattc
VPS29-5xFlag-Leu-2	*CATCTAATGTTTAGACATCATAGAAATGCATAAAAATGAAAATGGCTACC*ttaagcaaggattttcttaacttcttcggc
VPS35-5xFlag-Leu-1	*GTACATTGAAAGTCAAAGAGAAGTTGACGATCGTTTCAAAGTCATATATGTA*gactataaggatgatgacgacaaggaattc
VPS35-5xFlag-Leu-2	*GTAGTTTTTTTTATCTTGGGCATGTACGAAGAGCAAGTACGTTATTTAA*ttaagcaaggattttcttaacttcttcggc
VPS17-5xFlag-Leu-1	*CATCACTGAATGCGCGCCATGCTGCTTCACTTTTGGGCATGTCCACTAAA*gactataaggatgatgacgacaaggaattc
VPS17-5xFlag-Leu-2	*GGAAAAAGATCACCTTGTTCAAAGGTATGAATTTTCTACTTTATATACGTA*ttaagcaaggattttcttaacttcttcggc
VPS34-5xFlag-Leu-1	*CCTATCGTGATTGATCATTTACATAATCTGGCACAATACTGGCGGACC*gactataaggatgatgacgacaaggaattc
VPS34-5xFlag-Leu-2	*AATTAGAGTGACGAAATTTAAATTTTGAAGCACCAATTATCAACCAA*ttaagcaaggattttcttaacttcttcggc

[a] Italic capital letters represent homology arms.

Table S2. The 162 genes corresponding to $Na_2S_2O_5$-sensitive mutants identified from the genome-wide screen

Gene	ORF	Score 1	*p*-Value 1	Score 2	*p*-Value 2	Score 3	*p*-Value 3
VPS8	YAL002W	-0.8387	0	-0.72755	0.00025	-1.01233	0.00011
SWC3	YAL011W	-0.30217	0.00005	-0.21463	0.00002	-0.38179	0.00007
DRS2	YAL026C	-0.52512	0.00015	-0.59132	0.00005	-0.60141	0.00001
SLA1	YBL007C	-0.34975	0.00045	-0.50346	0.00005	-0.47773	0.0003
YBL094C	YBL094C	-0.27789	0.00007	-0.42182	0.00129	-0.63125	0.00119
MNN2	YBR015C	-0.24668	0.00001	-0.26021	0.00009	-0.35458	0.00004
RPS11B	YBR048W	-0.37053	0	-0.38711	0.00001	-0.40836	0.00024
SIF2	YBR103W	-0.24194	0.00002	-0.55185	0.00028	-0.36329	0.0001
PHO88	YBR106W	-0.40288	0.00002	-0.50536	0.00002	-0.60673	0.00005
AGP2	YBR132C	-0.28741	0.00002	-0.2621	0.00267	-0.30512	0.00043
YSW1	YBR148W	-0.43049	0.00005	-0.40755	0	-0.44093	0.00009
SMY2	YBR172C	-0.24527	0.02322	-0.27829	0.02968	-0.44074	0.04964
RPS6B	YBR181C	-0.22015	0.00003	-0.21685	0.0002	-0.26193	0.00005
MET8	YBR213W	-0.34369	0.00001	-0.42738	0.00001	-0.50046	0.00026
SNF5	YBR289W	-0.35079	0.00061	-0.3196	0.00172	-0.36101	0.00023
MAL32	YBR299W	-0.34019	0.00008	-0.32499	0.00001	-0.37155	0.00047
SRO9	YCL037C	-0.53916	0.00231	-0.44804	0.00034	-0.31166	0.00015
RVS161	YCR009C	-0.33163	0.00008	-0.39286	0.00115	-0.44876	0.00158
PER1	YCR044C	-0.36368	0.00004	-0.48635	0.00013	-0.3753	0.00008
YCR061W	YCR061W	-0.41786	0.00001	-0.33061	0.00025	-0.61145	0.00033
YCR062W	YCR062W	-0.30524	0.00001	-0.50999	0.00001	-0.36369	0.00001
PAT1	YCR077C	-0.45954	0.00018	-0.35966	0.00003	-0.27315	0.00017
RPN4	YDL020C	-0.37097	0.00001	-0.39768	0.00004	-0.38617	0.00002
ENT1	YDL161W	-0.59036	0.00003	-0.76398	0.00012	-0.64475	0.00001
UFD2	YDL190C	-0.45446	0.00023	-0.51681	0.00085	-0.44855	0.00067
OST4	YDL232W	-0.22667	0.00062	-0.43524	0.00038	-0.38986	0.00034
SNF11	YDR073W	-0.49468	0.00002	-0.3906	0.00004	-0.3535	0.00001
RPA14	YDR156W	-0.35858	0	-0.41551	0.00042	-0.29749	0.00014
SAC3	YDR159W	-0.97548	0.00235	-0.53011	0.00096	-0.83963	0.00008
RAV2	YDR202C	-0.46004	0.00017	-0.39697	0.00002	-0.34102	0
DPL1	YDR294C	-0.40126	0.00001	-0.4177	0.00003	-0.23496	0.00003
EAF1	YDR359C	-0.40934	0.00432	-0.27265	0.00002	-0.3474	0.00006
YDR360W	YDR360W	-0.23392	0.00004	-0.37669	0.00012	-0.2325	0.00002
STP1	YDR463W	-0.35197	0.00004	-0.23502	0	-0.47678	0.00009
RMT2	YDR465C	-0.44495	0.00035	-0.4439	0.00091	-0.55659	0.00212
PAC2	YER007W	-0.38217	0.0001	-0.41969	0.00025	-0.33901	0.00023
YER079W	YER079W	-0.44489	0.00007	-0.57604	0.00001	-0.45974	0.00036
GET2	YER083C	-0.39886	0.00006	-0.36588	0.00043	-0.5045	0.00023
UBP3	YER151C	-0.67235	0	-0.4699	0.00004	-0.32925	0.00002
BST1	YFL025C	-0.40768	0.00002	-0.63903	0.00057	-0.50143	0.00057
YGL007W	YGL007W	-0.56358	0.00001	-0.59857	0.00009	-0.56092	0.00021

GET1	YGL020C	-0.2991	0.00001	-0.29186	0.00001	-0.22727	0.00005
YGL024W	YGL024W	-0.59113	0.00251	-0.62375	0.00014	-0.56162	0.00002
YGL042C	YGL042C	-0.44979	0.00006	-0.54847	0	-0.60248	0.00043
DST1	YGL043W	-0.6467	0.0011	-0.90592	0.00116	-0.87742	0.00038
RIM8	YGL045W	-0.26007	0.00002	-0.31949	0.00001	-0.3382	0.00005
PMR1	YGL167C	-0.34807	0	-0.5439	0.00015	-0.45462	0
HUR1	YGL168W	-0.40319	0.00002	-0.53286	0.00011	-0.48714	0
SKI8	YGL213C	-0.57949	0.00129	-0.54281	0.00009	-0.43723	0.00004
YGL214W	YGL214W	-0.52834	0.00008	-0.47394	0.00005	-0.54563	0.00005
FZF1	YGL254W	-0.28545	0.00003	-0.21331	0.00001	-0.34556	0.00001
DBF2	YGR092W	-0.49425	0.00019	-0.65693	0.00382	-0.68679	0.00002
VMA21	YGR105W	-0.25816	0.00008	-0.27733	0.00001	-0.29103	0.00002
RPS23A	YGR118W	-0.33055	0.00005	-0.28099	0.00005	-0.20759	0.00001
SMI1	YGR229C	-0.33348	0.00006	-0.5016	0.00005	-0.51205	0.00003
RPL8A	YHL033C	-0.26956	0.00011	-0.26087	0.00002	-0.42835	0.00032
VPS29	YHR012W	-0.6728	0.00022	-0.58803	0.00001	-0.66176	0.00011
RPS27B	YHR021C	-0.21384	0	-0.26693	0.00012	-0.21704	0.00007
FYV4	YHR059W	-0.40063	0.00009	-0.29771	0.00001	-0.47814	0.00005
THP2	YHR167W	-0.22115	0.00002	-0.20627	0.0001	-0.40972	0.00002
SVP26	YHR181W	-0.21694	0	-0.2536	0	-0.26547	0.00001
CST6	YIL036W	-0.43367	0.00234	-0.41996	0.00074	-0.48685	0.00177
SER33	YIL074C	-0.7391	0.00271	-0.66207	0.00002	-0.75702	0.00012
RSM25	YIL093C	-0.42553	0.00012	-0.42267	0.00011	-0.70765	0.00094
ASG1	YIL130W	-0.30461	0.00013	-0.3266	0.00001	-0.57254	0.00011
MPH1	YIR002C	-0.7124	0.00004	-0.76965	0.00001	-0.79118	0.00001
DCG1	YIR030C	-0.28615	0.00001	-0.4792	0.00001	-0.85664	0.00007
MAD3	YJL013C	-0.73187	0.0001	-0.62916	0.00006	-0.86409	0
PEP8	YJL053W	-0.24694	0.00005	-0.22433	0.00029	-0.22816	0.00003
BNA3	YJL060W	-0.38717	0.00015	-0.4232	0.00003	-0.50539	0.00005
PBS2	YJL128C	-0.30155	0.0009	-0.48929	0.00075	-0.60533	0.00548
YJL199C	YJL199C	-0.39772	0.00023	-0.3684	0.00052	-0.5843	0.00826
RCY1	YJL204C	-0.47741	0.00002	-0.39845	0.00022	-0.5544	0.00007
RPL14A	YKL006W	-0.23426	0	-0.25436	0.00004	-0.59041	0.0005
MRT4	YKL009W	-0.23493	0.00046	-0.46381	0.00062	-0.41976	0.00019
GPX1	YKL026C	-0.47753	0.00024	-0.40233	0.00003	-0.61348	0.00168
AVT3	YKL146W	-0.35087	0.00144	-0.40718	0.00055	-0.59117	0.00518
RPS27A	YKL156W	-0.2288	0.00001	-0.30473	0.00002	-0.24702	0.00007
SAC1	YKL212W	-0.34184	0.00067	-0.38858	0.00036	-0.51208	0.00303
VPS51	YKR020W	-0.88792	0.0002	-0.73018	0.00006	-0.7093	0.00009
GCN3	YKR026C	-0.5609	0.00014	-0.61098	0.00033	-0.50889	0.00026
YKR035C	YKR035C	-0.31299	0.00004	-0.26869	0.0001	-0.79207	0.0002
DID2	YKR035W-A	-0.49202	0.00043	-0.44735	0.00154	-0.7118	0.00072
YLL044W	YLL044W	-0.20486	0.00004	-0.2671	0.00047	-0.31034	0.00131
RIC1	YLR039C	-0.41181	0.0002	-0.34539	0.00042	-0.42619	0.00186

SIC1	YLR079W	-0.20412	0.00082	-0.34723	0.0013	-0.33137	0.00004
SRN2	YLR119W	-0.58204	0.00015	-0.66158	0	-0.58481	0.00003
SWI6	YLR182W	-0.34648	0.00012	-0.55967	0.00032	-0.61373	0.00009
PBA1	YLR199C	-0.36557	0.00002	-0.37975	0.00005	-0.40318	0.00003
CPR6	YLR216C	-0.20059	0.00003	-0.26208	0	-0.32332	0.00006
CDA2	YLR308W	-0.397	0.00062	-0.27835	0.00019	-0.39023	0.00074
TMA10	YLR327C	-0.35828	0	-0.24841	0.00003	-0.3752	0
CHS5	YLR330W	-0.61346	0.00009	-0.88773	0.00017	-0.96357	0.00011
VRP1	YLR337C	-0.40474	0.0002	-0.58356	0.00005	-0.54718	0.00039
FKS1	YLR342W	-0.25086	0.00002	-0.31513	0.00009	-0.25173	0
VPS38	YLR360W	-0.32833	0	-0.26137	0.00019	-0.44751	0.00001
SKI2	YLR398C	-0.49772	0.00053	-0.48788	0.00181	-0.57739	0.00039
YLR402W	YLR402W	-0.73165	0.01558	-0.76532	0.00354	-0.73758	0.00087
PUN1	YLR414C	-0.42626	0.00002	-0.42982	0	-0.31976	0.00001
RPS1A	YLR441C	-0.33682	0	-0.2859	0.00002	-0.22064	0
ERG6	YML008C	-0.72043	0.00187	-0.7212	0.0011	-0.60234	0.00005
GSF2	YML048W	-0.55126	0.00012	-0.63181	0.00003	-0.37857	0.00015
RPS1B	YML063W	-0.64245	0.00004	-0.58029	0.00028	-0.70543	0.00004
VPS9	YML097C	-0.33538	0.00001	-0.24923	0.00021	-0.3148	0.00001
YMR007W	YMR007W	-0.41339	0.0003	-0.38328	0.00056	-0.26653	0.00013
YMR031W-A	YMR031W-A	-0.78587	0.00002	-0.78764	0.00021	-0.78628	0.00006
RIM9	YMR063W	-0.36114	0.00021	-0.27362	0.00001	-0.34843	0.00003
REC114	YMR133W	-0.44218	0.00023	-0.43229	0.00028	-0.34798	0.00006
NDE1	YMR145C	-0.52084	0	-0.7071	0.00008	-0.90558	0.00021
ERG2	YMR202W	-0.47992	0	-0.48513	0.00012	-0.42874	0
RPS10B	YMR230W	-0.44721	0	-0.36901	0	-0.25158	0.00004
SAP30	YMR263W	-0.36823	0.00003	-0.42475	0.00017	-0.40827	0.00003
TMA23	YMR269W	-0.26411	0	-0.38249	0.00001	-0.46179	0.00005
FKS3	YMR306W	-0.464	0.00006	-0.53406	0.00045	-0.6055	0.00144
GAS1	YMR307W	-0.23959	0	-0.44151	0.00004	-0.49668	0.00007
YNL040W	YNL040W	-0.36855	0.00001	-0.26125	0.00005	-0.25918	0.00005
RPL9B	YNL067W	-0.36098	0.00002	-0.41209	0.00021	-0.49545	0.00003
EOS1	YNL080C	-0.4905	0.00004	-0.49137	0.00026	-0.5426	0.00075
YNL089C	YNL089C	-0.47844	0.00001	-0.35125	0	-0.4463	0
NST1	YNL091W	-0.36045	0.00039	-0.37878	0.00038	-0.49962	0.00038
RPS7B	YNL096C	-0.30839	0.00012	-0.34252	0.00009	-0.39891	0.00015
PHO23	YNL097C	-0.37491	0.00077	-0.26328	0.00051	-0.62247	0.01842
ESBP6	YNL125C	-0.23634	0.00001	-0.25695	0.00012	-0.36427	0.00008
FPR1	YNL135C	-0.45397	0.00002	-0.29202	0.00001	-0.37156	0.00008
YNL143C	YNL143C	-0.24848	0.00001	-0.28511	0.0001	-0.51486	0.00013
YNL198C	YNL198C	-0.23327	0.00004	-0.47712	0.00002	-0.45539	0
ZWF1	YNL241C	-0.2981	0.00001	-0.38539	0.00026	-0.32829	0
GIS2	YNL255C	-0.61942	0.00038	-0.45583	0.00055	-0.45552	0.00003
RIM21	YNL294C	-0.23119	0.00001	-0.27922	0	-0.35501	0

YNR005C	YNR005C	-0.51462	0	-0.22671	0.00008	-0.56811	0.00003
FPK1	YNR047W	-0.36188	0.00003	-0.2561	0	-0.43102	0.00002
BRE5	YNR051C	-0.52596	0.00016	-0.3839	0.00001	-0.50534	0.00016
TLG2	YOL018C	-0.21188	0.00032	-0.26892	0.0001	-0.36391	0.0002
RPP2A	YOL039W	-0.39553	0.00003	-0.26998	0.00002	-0.37932	0
NTG2	YOL043C	-0.55608	0.00003	-0.32854	0.00001	-0.41099	0.00057
PSK2	YOL045W	-0.35477	0.00024	-0.2409	0.00003	-0.46336	0.0001
DUF1	YOL087C	-0.27433	0.00001	-0.20144	0.00002	-0.32966	0.00001
YOL114C	YOL114C	-0.20172	0.00067	-0.22367	0.0004	-0.38892	0.00073
YOL118C	YOL118C	-0.28317	0.00001	-0.21997	0.00003	-0.26863	0.00002
TSR3	YOR006C	-0.44269	0.00021	-0.45835	0.00008	-0.43004	0.00003
SGT2	YOR007C	-0.47356	0.01737	-0.36791	0.01535	-0.63253	0.01311
CKB2	YOR039W	-0.28694	0	-0.20482	0.00001	-0.37589	0.00002
VAM10	YOR068C	-0.5696	0.00023	-0.58333	0.00011	-0.72369	0.00026
VPS5	YOR069W	-0.41265	0.0002	-0.39007	0.00002	-0.44824	0.00485
GYP1	YOR070C	-0.48973	0.00001	-0.46511	0.00003	-0.54974	0.00019
SKI7	YOR076C	-0.52843	0.00004	-0.50585	0.00002	-0.48722	0.00021
LPX1	YOR084W	-0.29115	0.00003	-0.22642	0.00001	-0.22803	0.00035
OST3	YOR085W	-0.2393	0.00008	-0.45115	0	-0.4365	0.00006
VAM3	YOR106W	-0.24833	0.00005	-0.32807	0.00009	-0.27039	0.00003
GCY1	YOR120W	-0.23099	0	-0.30105	0.00001	-0.32566	0.00001
YOR135C	YOR135C	-0.64582	0.00006	-0.54642	0	-0.70305	0.0001
MRS2	YOR334W	-0.3021	0.00005	-0.3857	0.00008	-0.45548	0.0002
SNX3	YOR357C	-0.51634	0.00176	-0.53629	0.00201	-0.59044	0.00418
SSU1	YPL092W	-0.85768	0.00192	-0.927	0.00028	-0.88724	0.00046
VPS30	YPL120W	-0.42422	0.00112	-0.45241	0.00572	-0.44048	0.0022
TGS1	YPL157W	-0.5319	0.00042	-0.75115	0.00042	-0.92181	0.00108
THI6	YPL214C	-0.68115	0.00008	-0.76927	0.00003	-0.75936	0.00003
ACM1	YPL267W	-0.32866	0.00001	-0.21508	0	-0.22805	0.00004
TKL1	YPR074C	-0.21339	0.00051	-0.2282	0.00056	-0.25944	0.00241
YPR084W	YPR084W	-0.32035	0.00001	-0.31864	0	-0.42614	0.00001
VPS4	YPR173C	-0.75536	0.00039	-0.6615	0.00004	-0.80291	0.00039
SKI3	YPR189W	-0.49033	0.00018	-0.52781	0.0001	-0.44907	0.00005

Table S3. The 16 genes corresponding to $Na_2S_2O_5$-tolerant mutants identified from the genome-wide screen

Gene	ORF	Score 1	*p*-Value 1	Score 2	*p*-Value 2	Score 3	*p*-Value 3
FLO11	YIR019C	0.21199	0.00006	0.22818	0.00003	0.36131	0.00005
FUM1	YPL262W	0.37151	0	0.38594	0	0.49737	0.00001
INH1	YDL181W	0.41922	0	0.28602	0	0.32748	0.00004
LYS20	YDL182W	0.22803	0.00001	0.20714	0.00001	0.27348	0.0001
MMS22	YLR320W	0.28691	0.00001	0.20887	0.00001	0.21211	0
MRE11	YMR224C	0.3067	0	0.33926	0.00028	0.33313	0.00002
NTG1	YAL015C	0.2424	0	0.65947	0	0.22463	0.00001
PHO86	YJL117W	0.20082	0.00002	0.23839	0.00001	0.26717	0
PYK2	YOR347C	0.20441	0.00003	0.41727	0.00001	0.31334	0
RAD23	YEL037C	0.22203	0.00001	0.29977	0.00001	0.35245	0.00003
RPL40B	YKR094C	0.46074	0.00001	0.41839	0	0.32407	0
SFH5	YJL145W	0.21624	0.00004	0.30368	0.00008	0.28467	0.00017
VID24	YBR105C	0.2094	0	0.25375	0.00009	0.42378	0.00023
MRX19	YDL183C	0.29817	0.00001	0.20719	0.00001	0.24431	0
YDR248C	YDR248C	0.31247	0.00005	0.21664	0.00007	0.50528	0.00002
YNL120C	YNL120C	0.52946	0.00005	0.29693	0.00001	0.23207	0.00002
